# The instrumental role of lipids in governing the sensitivity of multiple myeloma to ferroptosis

**DOI:** 10.1007/s12672-025-03444-9

**Published:** 2025-08-25

**Authors:** Ali Habib, Oliver G. Best, Charlotte E. Toomes, Craig T. Wallington-Gates

**Affiliations:** 1 College of Medicine and Public Health, Flinders University, Bedford Park, SA 5042 Australia; 2 School of Health, University of the Sunshine Coast, Sippy Downs, QLD 4556 Australia; 3 School of Medicine and Dentistry, Griffith University, Birtinya, QLD 4575 Australia; 4 Department of Haematology, Sunshine Coast University Hospital, Birtinya, QLD 4575 Australia

**Keywords:** Cancer, Haematology, Multiple myeloma, Ferroptosis, Lipids, Phospholipids, Cholesterol, Fatty acids, Saturated, Unsaturated

## Abstract

Multiple myeloma (MM) is a malignancy characterised by the uncontrolled proliferation of clonal plasma cells, primarily within the bone marrow, and is still considered incurable. A significant proportion of patients relapse with drug-refractory disease, necessitating the development of novel therapeutic approaches. Ferroptosis is a recently-characterised form of non-apoptotic programmed cell death, linked to phospholipid peroxidation, that represents a promising approach for the treatment of MM and other cancers, that are refractory to more conventional apoptosis-inducing regimens. A better understanding of the relationship between cellular lipid composition and ferroptosis sensitivity is key to harnessing this form of programmed cell death as a therapeutic approach. In addition to the cellular proportions of phospholipids containing poly- and monounsaturated fatty acids, studies to date indicate that cholesterol levels impact not only the onset and progression of haematological malignancies but also the sensitivity of a variety of different cancers to ferroptosis. Therefore, manipulating the uptake and metabolism of lipids, including glycerophospholipids and cholesterol, may be an effective means of sensitising MM cells to ferroptosis. Findings from the limited number of studies concerning ferroptosis in MM and compelling evidence from other malignancies, provide a strong rationale for further investigation of ferroptosis as a novel therapeutic approach for MM.

## Background

Multiple myeloma (MM) is a malignancy characterised by the uncontrolled proliferation of clonal plasma cells, primarily within the bone marrow, and is still considered incurable [1, 2]. MM is the second most common haematological malignancy after non-Hodgkin lymphoma, with > 175,000 new cases diagnosed annually worldwide [3], including approximately 2,600 Australians [4]. Clinical features of MM include hypercalcaemia, renal insufficiency, anaemia, bone lesions, and bone marrow failure. Until the turn of the century, 5-year survival rates among MM patients were as low as 25% [5] and despite advances in treatment, the 5-year survival rate remains around 50%, with a median overall survival of 5.5 years [6]. Furthermore, the 5-year progression-free survival rate is only 17% for patients deemed to have high-risk disease [7] and survival among older MM patients (> 65 years) has stagnated over the last 20 years [6]. Novel therapeutic approaches are urgently needed to improve outcomes for MM patients.

The median age of diagnosis for MM is 70 years and therefore, frailty and the presence of comorbidities often limit the use of intensive therapies [4]. There is also an increasing awareness of patients with ‘functional high-risk’ myeloma who exhibit poor responses to therapy or experience rapid relapses that are independent of genetic risk factors. Over the past two decades, the standard of care for MM patients has moved beyond conventional chemotherapy to include proteasome inhibitors (PIs - bortezomib, carfilzomib, ixazomib, etc.), immunomodulatory drugs (IMiDs - thalidomide, lenalidomide, pomalidomide, etc.) and monoclonal antibodies (mAbs – daratumumab (anti-CD38), isatuximab (anti-CD38), elotuzumab (anti-SLAMF7), etc.). Other immune and cell-based therapeutic approaches are also emerging, including bi-specific antibodies (BsAbs – teclistamab, elranatamab, etc.), antibody-drug conjugates (ADCs – belantamab mafodotin) and chimeric antigen receptor T cell therapies (CAR-T – ciltacabtagene autoleucel (cilta-cel), idecabtagene vicleucel (ide-cel)) [8].

The efficacy of many of the cancer therapy regimens currently in use rely on the induction of cell death. Cell death is a fundamental biological process that ensures that the integrity and function of cells and tissues are maintained, thereby preventing tumorigenesis. Cell death can generally be classified as either unprogrammed or programmed cell death (PCD) [9]. Unprogrammed cell death occurs in a non-regulated manner, typically in response to overwhelming chemical or physical stimuli [9, 10]. In contrast, PCD is a tightly regulated process that is essential for tissue homeostasis and protection against viruses and disease [10]. Various mechanisms of PCD have been identified, including apoptosis, autophagy, pyroptosis, necroptosis and ferroptosis [10]. This review will focus on our current knowledge regarding ferroptosis, its emerging role as an important PCD mechanism, its association with lipids, and how ferroptosis may represent a novel approach for the treatment of MM.

### Ferroptosis

Ferroptosis is an iron-dependent, non-apoptotic form of PCD, characterised by lipid peroxidation [11]. Ferroptosis is distinct from other forms of PCD in terms of the morphological changes that occur and molecular mechanisms that drive cell death, illustrated by evidence that inhibitors of apoptosis have little or no effect on ferroptosis-mediated cell death [11]. Interest in ferroptosis-mediated therapeutic strategies has grown significantly in recent years, as these may represent a means of overcoming resistance to more conventional therapies that are generally reliant on apoptosis.

**Fig. 1:**
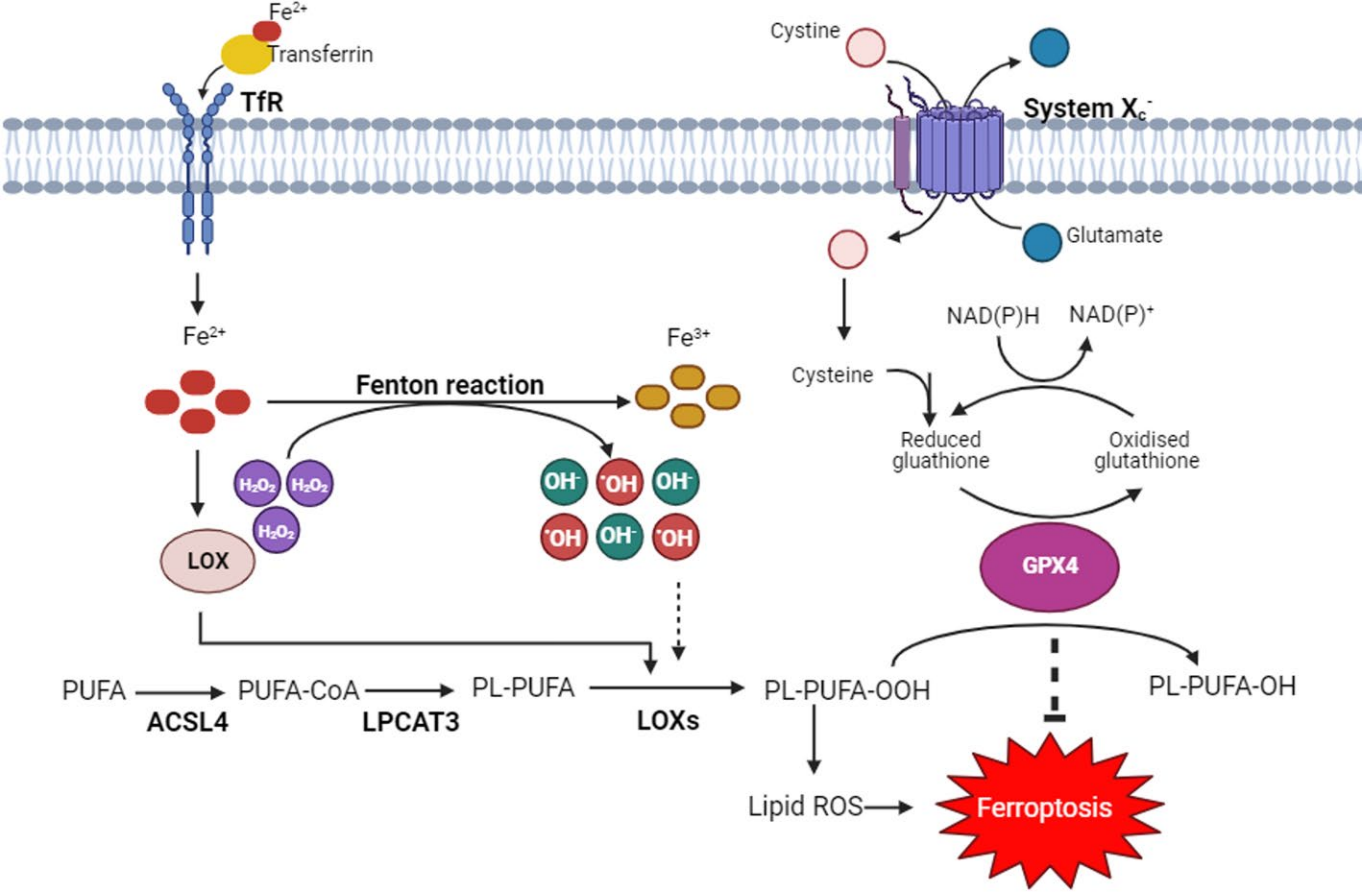
Ferroptosis, driven by lipid peroxidation, is inhibited by system X_c_^-^ and GPX4.

ACSL4, acyl-CoA synthetase long-chain family member 4; Fe^2+^, ferrous iron; Fe^3+^, ferric iron; GPX4, glutathione peroxidase 4; H_2_O_2_, hydrogen peroxide; LOXs, lipoxygenases; LPCAT3, lysophosphatidylcholine acyltransferase 3; OH^−^, hydroxide ion; .OH, hydroxyl radical; PL, phospholipid; PL-PUFA-OH, phospholipid alcohol; PL-PUFA-OOH, phospholipid hydroperoxides; PUFA, polyunsaturated fatty acid; ROS, reactive oxygen species; TfR, transferrin receptor. Created with BioRender.com.

Lipid peroxidation is a hallmark of ferroptosis and is characterised by reactions that result in the oxidative degradation of lipids, yielding highly toxic peroxyl radicals (ROO•) [12]. Oxidation and subsequent degradation of lipids, which is catalysed by lipoxygenase enzymes (Fig. 1), results in the formation of peroxyl radicals that irreversibly compromise the integrity of cellular membranes leading to cell death [13]. However, cancer cells adapt and can develop mechanisms to buffer the harmful effects of lipid peroxidation and reactive oxygen species (ROS) that accumulate due to the high metabolic demands and rapid proliferation of the tumour.

Iron is an important co-factor for lipoxygenases and is essential for the initiation and promotion of lipid peroxidation via the Fenton reaction [14]; the importance of iron and lipid peroxidation in ferroptosis is demonstrated by evidence that both iron chelators (deferoxamine) and synthetic antioxidants (e.g. liproxstatin-1 and ferrostatin-1) can inhibit this form of cell death by binding free iron and scavenging free radicals, respectively [15].

### Inhibition of ferroptosis

#### System X_c_^-^ and glutathione peroxidase 4 (GPX4)

Plasma membrane-bound system X_c_^−^ is an important regulator of ferroptosis. This antiporter system facilitates the exchange of intracellular glutamate for extracellular cystine, which is then rapidly converted to cysteine by the thioredoxin reductase 1 (TXNRD1) enzyme (Fig. 1) [16]. Cysteine is the rate limiting step in the biosynthesis of the antioxidant and enzyme substrate, glutathione (GSH). GSH activates the catalytic domain of the selenoprotein, glutathione peroxidase 4 (GPX4) [16], which converts toxic lipid peroxides into neutral alcohols, thereby inhibiting lipid peroxidation and ferroptosis (Fig. 1) [16, 17].

### The role of antioxidants in modulating ferroptosis via FSP1 and the mevalonate pathway

**Fig. 2:**
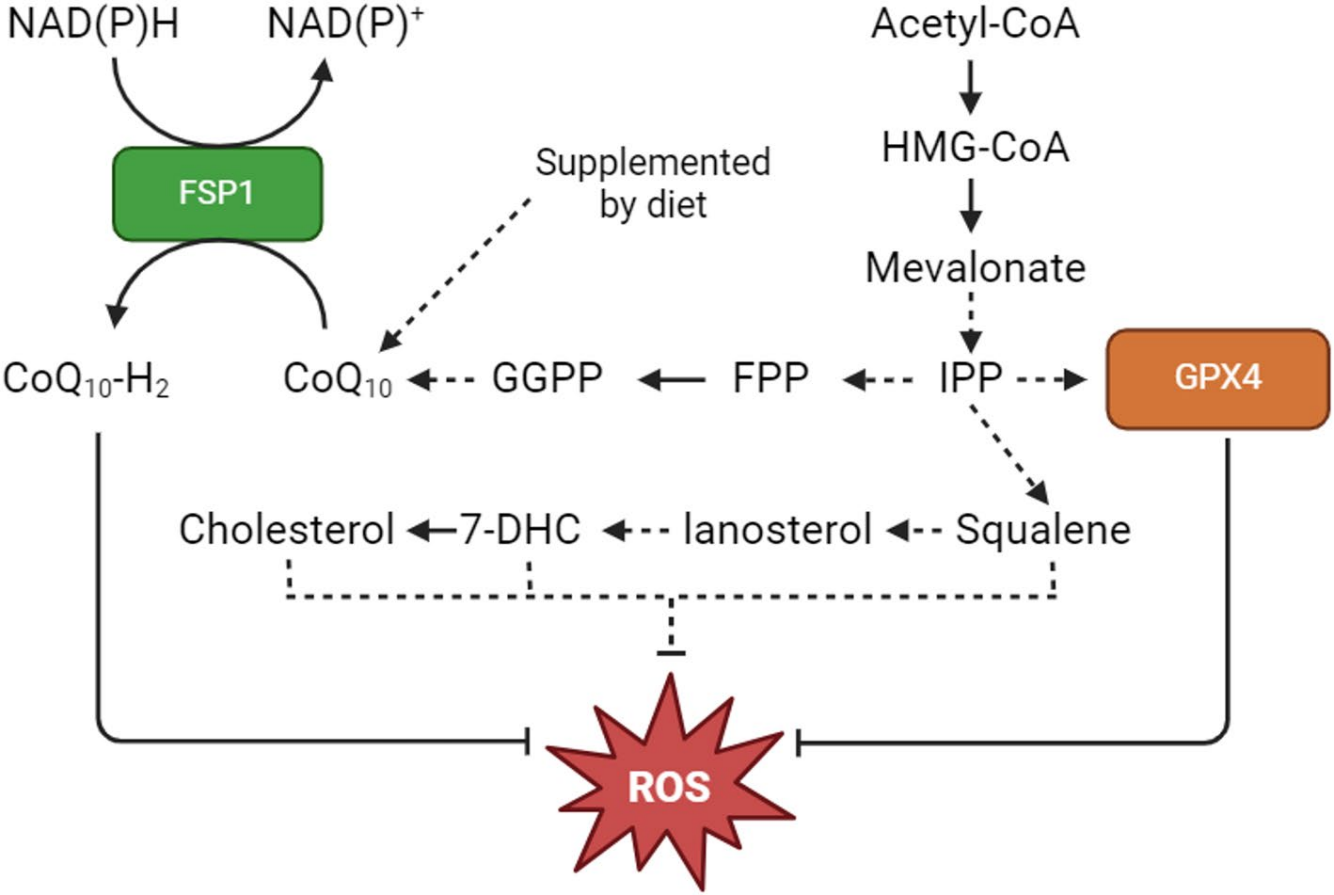
Biochemical pathways of the mevalonate pathway in the inhibition of ferroptosis.

GPX4-independent antioxidant systems also play an important role in suppressing ferroptosis. One such system is the ferroptosis suppressor protein 1 (FSP1)/mevalonate pathway [18], which is involved in the production of isopentenyl pyrophosphate (IPP) and coenzyme Q10 (CoQ10) (Fig. 2). This involves the reduction of acetyl-CoA to mevalonate and subsequent conversion to IPP [19], which in turn is converted to the CoQ10 substrate, farnesyl pyrophosphate [19]. Farnesyl pyrophosphate is also involved in the maturation of selenocysteine, an amino acid required for translation of GPX4 [20, 21]. CoQ10 is a naturally occurring quinone that is vital to cell and tissue health in most aerobic organisms [22]. CoQ10 is primarily involved in the mitochondrial electron transport chain, where it functions as a high-energy transfer molecule [22]. The biosynthesis of CoQ10 begins and ends in the mitochondria and is facilitated by a complex of proteins, which have not yet been fully elucidated [23].

CoQ10 is comprised of a benzoquinone ring derived from the amino acid tyrosine, which is chemically linked to 10 isoprenoid units and synthesised by the mevalonate pathway [24]. Ferroptosis suppressor protein 1 (FSP1) catalyses the regeneration of CoQ10 into its reduced form, CoQ_10_-H_2_ (ubiquinol), which functions to trap radicals (Fig. 2) [18]. FSP1 is not involved in the canonical ferroptosis pathway but does protect cells against ferroptosis-inducing agents [18]; expression of FSP1 has been shown to correlate with sensitivity to ferroptosis-inducing compounds, including RSL3, while genetic knockdown of *FSP1* has been shown to sensitise a range of different cancer cell lines to ferroptosis-inducing compounds [18]. Furthermore, FSP1 does not protect cells against pro-apoptotic agents and is not regulated by the tumour suppressor protein, TP53.

7-DHC, 7-Dehydrocholesterol; Acetyl-CoA, acetyl coenzyme A; CoQ10, ubiquinone; CoQ10-H2, ubiquinol; FPP, farnesyl phosphate; FSP1, ferroptosis suppressor protein 1; GGPP, geranylgeranyl pyrophosphate; GPX4, glutathione peroxidase 4; HMG-CoA, 3-hydroxy-3-methylglutaryl-coenzyme A; IPP, isopentenyl phosphate; ROS, reactive oxygen species. Dotted arrows represent multiple steps within a pathway. Created with BioRender.com.

### The role of lipids in ferroptosis

#### Glycerophospholipids

Lipids are a diverse group of organic molecules that are essential building blocks of life; they make up the structural composition of cell membranes, play important roles as signalling molecules and are a critical source of energy [25]. Lipids can be loosely categorised into fatty acids (FAs), glycerides, non-glyceride lipids and complex lipids. In the context of ferroptosis, phospholipids (PLs), specifically glycerophospholipids, play important roles in the initiation and propagation of lipid peroxidation (Fig. 1) [25].

FAs are carboxylic acids with an aliphatic chain, comprised of oxygen, carbon, and hydrogen atoms. They can be categorised into three distinct groups, saturated fatty acids (SFA), monounsaturated fatty acids (MUFA) and polyunsaturated fatty acids (PUFA) [26, 27]. PLs, particularly those that are comprised of PUFAs, are pivotal in ferroptosis, since they are more readily oxidised than other lipids. Emerging research in the ferroptosis space has also suggested that the degree of PL acyl chain saturation correlates with ferroptosis sensitivity [28]. The intracellular mechanisms that inhibit ferroptosis once lipid peroxidation has been initiated are complex and several pathways are now known to be involved, including the antioxidant systems coordinated by FSP1 and GPX4 [29].

There is a growing body of literature concerning the role of PL-PUFA in ferroptosis, highlighting the association between the lipid composition of different cell types and their sensitivity to ferroptosis. Several studies have examined the effects of exogenous PUFAs, including arachidonic (AA), eicosapentaenoic and docosahexaenoic acid, on cancer cells or cardiomyocytes in vitro (Table 1). Recent research has demonstrated that t(4;14)-positive MM models, a poor prognostic abnormality in MM, are sensitive to ferroptosis induced by class II ferroptosis inducers in vitro and in vivo [30]. The study found that the upregulation of MM SET domain-containing protein (MMSET) in these models resulted in the upregulation of ACSL4 (acyl-CoA synthetase long-chain family member 4), in turn increasing cellular PUFA levels [30]. A study published by our group has demonstrated that delivering PUFA to MM cells via liposomes, which are lipid-based nanoparticles, can sensitise them to the GPX4 inhibitor RSL3 [31]. These studies demonstrate that by increasing the intracellular proportion of these FAs, cells can be sensitised to ferroptosis and that in some cell types, this alone was sufficient to induce ferroptosis [27, 32, 33]. In contrast, increased levels of PLs containing MUFA (PL-MUFA) or other less readily oxidised lipid species (PL-SFA) within the lipidome have been shown to increase the resistance of cells to ferroptosis (Table 1) [16, 21, 34]. Furthermore, there is evidence that MUFAs can displace PUFAs from PLs in the lipid membrane and other subcellular regions [34].

There is also evidence that SFAs may play a role in ferroptosis, but the findings of these studies are inconsistent, with some studies suggesting that SFAs play an important role in lipotoxicity, and not ferroptosis [35]. However, another study suggested that the SFA, palmitic acid, is actively involved in lipid ROS production and ferroptosis in insulin-producing β-cells [36]. GPX4 overexpression and ferrostatin-1 were able to partially prevent cell death, while co-treatment of the cells with the MUFA, oleic acid, protected β-cells from lipid peroxidation induced by palmitic acid [36]. Magtanong et al.., also found that culturing cells with exogenous MUFA, but not treatment with ferrostatin-1, was protective against palmitic acid-induced cell death, suggesting that ferroptosis may not be the primary mechanism of cell death [34].

Two other studies of cardiomyocytes and colorectal cancer cell lines support the finding that palmitic acid can induce lipid peroxidation but suggest that this effect, and subsequent ferroptosis, is dependent on expression of the lipid transporter, CD36, in the plasma membrane [37, 38]. In a similar study, Kuang et al.., suggested the effects of palmitic acid on ferroptosis in colon cancer cells were due to induction of non-canonical ferroptosis via CD36, leading to an increase in ER stress, endocytosis of transferrin and an increase in intracellular ferrous iron levels [38]. In contrast, another study showed that the SFA, stearic acid, can protect cardiomyocytes from ferroptosis, which may be due to the fact this FA is less readily oxidised than the PUFAs it displaces in the lipidome [39]. Collectively, these studies suggest that the role(s) of SFAs in ferroptosis are complex and probably context-dependent and although SFAs may have more significant roles in lipotoxicity, there is evidence that SFAs can act as either inhibitors or promoters of ferroptosis.

#### Lysophospholipids

Lysophospholipids (LPL) represent a relatively minor component of the total lipid composition in cells and are characterised by a polar head group and a single carbon (acyl) chain. The lysolipid structure within LPLs confers hydrophilicity and versatility, which facilitates their many tissue specific functions [40]. LPLs are recognised as extracellular, and in some cases intracellular, signalling mediators [40]. LPLs are commonly formed when the ester bonds of PLs are enzymatically hydrolysed by phospholipase A, producing LPL and a free fatty acid chain [41].

LPL levels have been found to be significantly elevated in cells undergoing ferroptosis, with a concomitant decrease in the corresponding PL-PUFA [42, 43]. This is thought to be due to oxidised acyl chains being the preferred substrate for specific lipases, particularly phospholipase A [43]. Inhibition of phospholipase A can rescue GPX4 null cells from ferroptosis, suggesting cleaved and oxidised PUFAs are actively involved in ferroptosis-associated membrane damage and cell death [43]. These results also suggest that increased levels of LPL may be an indication of ferroptosis-mediated cell death.

### Cholesterol

Cholesterol is a major component of cell membranes and is comprised of a hydrocarbon tail, a central sterol nucleus, and a hydroxyl group [44]. Cholesterol provides membrane stability and fluidity, is important for the formation of lipid rafts, and is a crucial precursor in the synthesis of steroid hormones, vitamin D and bile acids [44]. Cholesterol may also play an important role in cancer progression by promoting proliferation, invasion, and migration [45], and may impact the sensitivity of tumour cells to ferroptosis [46, 47]. Elevated levels of intracellular cholesterol have been shown to reduce the sensitivity of human epithelial cells to ferroptosis by increasing squalene and CoQ10, with effects similar to those observed with ferrostatin-1 [46]. This study went on to demonstrate that cholesterol inhibits ferroptosis in induced liver injury in mice [46]. Squalene is a lipophilic metabolite formed during the conversion of farnesyl pyrophosphate to either cholesterol through the mevalonate pathway or oxysterol via the squalene dioxide pathway [48]. Squalene protects cells from oxidative stress by altering the intracellular lipid composition and reducing ROS levels [48, 49]. Given the roles of squalene, the enzyme squalene synthase is now recognised as a potential therapeutic target for inducing ferroptosis [48].

A recent study revealed that long-term hematopoietic stem cells (HSCs) from C57BL/6 mice fed a high-cholesterol diet had significantly lower levels of lipid peroxidation and Fe^2+^ and increased levels of glutathione compared to controls [50]. Consistent with these changes, the cells were significantly less sensitive to erastin ex vivo. Elevated levels of IL-3 and GM-CSF induced by the high cholesterol diet al.so increased activity of the mTOR-mediated signalling pathway and subsequent upregulation of SLC7A11/GPX4 expression in the HSCs [50].

Cholesterol is also crucial for the formation of lipid rafts which are important components of the plasma membrane, coordinating cell signalling and import and export from the cell, in addition to regulating membrane fluidity and organisation [51]. Lipid rafts also impact the sensitivity of cells to ferroptosis [52]. A study found that cholesterol accumulation protects different cell types from ferroptosis by increasing the number of lipid rafts and by reducing the effects of lipid peroxidation on membrane fluidity and permeability [51]. RSL3 treatment in human melanoma and renal cancer cell lines resulted in an increase in lipid raft formation, while the combination of LDL and RSL3 yielded the densest membrane rafts, representing a dynamic shift in the lipidome in response to ROS build up [51]. Conversely, cholesterol depletion increased the sensitivity of the cells to ferroptosis-mediated cell death by decreasing lipid rafts and increasing membrane fluidity [51]. Similar results were also observed in vivo in a melanoma mouse model treated with GPX4 inhibitor, ML210, with a concomitant increase in lipid raft formation in xenograft derived cells post-treatment [51]. In a subsequent study, Zhao et al., determined that the increase in lipid raft density in response to GPX4 inhibition was mediated by ACSL4 (acyl-CoA synthetase long-chain family member 4) and that this was associated with a reduction in the efficacy of platinum-based drugs [53]. These results suggest that cancer cells can increase ferroptosis resistance through active modulation of the plasma membrane, in response to GPX4 inhibition.

In a study by Bai et al.., nanozymes capable of depleting cholesterol and disrupting lipid rafts were tested against a breast cancer cell line resulting in an increase in membrane fluidity and ferroptosis sensitivity of the cancer cells, via a mechanism involving downregulation of both GPX4 and FSP1 [54]. Similar results were observed in vivo, with evidence suggesting the efficacy of the nanozymes was mediated by both ferroptosis and anti-tumour immune responses [54].

Statins, a class of medications that inhibit 3-hydroxy-3-methylglutaryl-coenzyme A (HMG-CoA) reductase (a key enzyme in cholesterol synthesis) thereby reducing cholesterol synthesis, have also been implemented in ferroptosis [55]. A study in tripe-negative breast cancer has demonstrated that simvastatin containing nanoparticles could induce ferroptosis in vitro and could significantly reduce tumour volume in MDA-MB-231 tumour-bearing mice [56]. Another study has shown that simvastatin can induce ferroptosis in gastric cancer cells by inhibiting programmed cell death ligand 1 [57]. These results were further supported in nude mice, where simvastatin induced ferroptotic cell death, reducing both the weight and proliferation of subcutaneous tumours [57]. These studies suggest that statins, which are amongst the most frequently prescribed medications worldwide, can be used to combat cancer through the induction of ferroptosis.

**Table 1:** Lipids and their role in ferroptosis.

Lipid	Cancer/Cell Type	Effects	Mechanism	References
Arachidonic acid (PUFA)	Multiple myeloma, colorectal cancer, cervical cancer, hypopharyngeal cancer, melanoma, mouse melanoma and Lewis lung cancer	Promotes ferroptotic cell death	Serve as fuel for lipid peroxidation, due to ability to be readily oxidised by ROS and potent radicals	[58–60]
Docosahexaenoic acid (PUFA)	Multiple myeloma, prostate cancer, colorectal cancer, cervical cancer, hypopharyngeal cancer and liver cancer	Promotes ferroptotic cell death	Serve as fuel for lipid peroxidation, due to ability to be readily oxidised by ROS and potent radicals	[31, 32, 60, 61]
Eicosapentaenoic acid (PUFA)	Colon cancer, colorectal adenocarcinoma, cervical cancer, hypopharyngeal cancer, melanoma, carcinoma, osteosarcoma, Multiple Myeloma and hepatoma	Promotes ferroptotic cell death	Serve as fuel for lipid peroxidation, due to ability to be readily oxidised by ROS and potent radicals	[60–63]
Linoleic acid (PUFA)	Pancreatic cancer, melanoma, carcinoma ovarian cancer and hepatoma	Promotes ferroptotic cell death	Serve as fuel for lipid peroxidation, due to ability to be readily oxidised by ROS and potent radicals	[62, 64]
Oleic acid (MUFA)	Melanoma and ovarian cancer	Protects against ferroptosis	Less susceptible to oxidation and can displace PUFA in the lipid membrane	[65, 66]
Palmitoleic acid (MUFA)	Oesophageal squamous cell carcinoma and ovarian cancer	Protects against ferroptosis	Less susceptible to oxidation and can displace PUFA in the lipid membrane	[66, 67]
Stearic acid (SFA)	Cardiomyocytes	Protects against ferroptosis	Less susceptible to oxidation	[39]
Palmitic (SFA)	Colon cancer and cardiomyocytes	Promotes ferroptotic cell death	Induces ferroptosis via CD36, activating ER stress and breaking calcium-iron balance	[37, 38]
Coenzyme Q10 (Ubiquinone)	Colon, breast, lung, pancreas, brain, liver, kidney, skin and intestinal cancer cell lines	Prevents cellular accumulation of ROS and ferroptotic cell death	Powerful antioxidant that can inhibit lipid peroxidation	[18, 24]
Squalene	Anaplastic large cell lymphoma, fibrosarcoma and renal adenocarcinoma	Prevents cellular accumulation of ROS and ferroptotic cell death	Free radical scavenger and actively involved in cholesterol biosynthesis as a precursor	[46, 49]
Cholesterol	Multiple myeloma, fibrosarcoma, mouse liver and renal adenocarcinoma	Protects against ferroptosis	Important in maintaining cellular membrane integrity while also inhibiting lipid peroxidation	[46, 47]

### The role of the cholesterol synthesis pathway in ferroptosis: implications for therapy of cancers, including multiple myeloma

Despite significant advances in the treatment of many cancers, disease relapse and drug resistance remain the most challenging aspects of patient management. Since the efficacy of many current therapies relies on apoptosis-mediated cell death, induction of ferroptosis may represent an effective alternate therapeutic approach [68, 69]. However, the role of ferroptosis in the pathobiology of cancers and the mechanisms related to the sensitivity of cancers to this form of programmed cell death, are yet to be fully elucidated [70]. Given growing evidence that cholesterol and its precursors are important modulators of ferroptosis, the various pathways involved in lipid metabolism, including the cholesterol biosynthesis pathway, have been proposed as promising targets for ferroptosis-mediated therapeutics [46, 50]^71^– [75]. The next section of this review will focus on studies concerning the impact of cholesterol biosynthesis on the ferroptosis sensitivity of cancer cells, with a focus on MM.

### Cholesterol

Cholesterol has long been linked to tumorigenesis, cancer progression and treatment resistance [76–78]. MM patients have been reported to have significantly lower levels of serum cholesterol compared to healthy individuals and low cholesterol levels have been associated with a higher risk of MM onset [79]. The importance of lipids in ferroptosis suggests that cholesterol also likely impacts the sensitivity of MM cells to this form of cell death. However, further studies are required to more clearly elucidate the relationship between cholesterol levels and ferroptosis sensitivity in MM.

A population study of 3,500,000 individuals found that low levels of high-density lipoprotein cholesterol (HDL-C) were associated with an increased risk of MM [79]. The study proposed that the anti-inflammatory properties of HDL-C may protect against MM tumorigenesis and that low serum levels of HDL-C may be due to increased uptake and metabolic changes within the malignant plasma cells [79]. However, contrary to previous studies [75, 80], the data suggested that total serum cholesterol levels were similar between MM and healthy individuals. Another study of 502,507 participants in the United Kingdom found that higher levels of total cholesterol, HDL-C, and low-density lipoprotein cholesterol (LDL-C) were associated with a decreased risk of MM and other plasma cell neoplasms [81].

Both studies were conducted on peripheral blood plasma and levels of cholesterol in plasma cells were not assessed. Therefore, it is not possible to ascertain from these studies whether the decreased plasma cholesterol levels among the MM patients were associated with increased uptake into the tumour cells. Another study investigating apoptosis in MM found that addition of exogenous LDL to MM cells cultured in delipidated serum, increased their viability, while cholesterol depletion triggered an increase in the expression of LDL receptors [82]. The anti-apoptotic effects of cholesterol were also evident in primary MM cells, but not their healthy counterpart [82]. While the focus of the study by Tirado-Velez et al. [82]. , was apoptosis rather than ferroptosis, it does suggest that uptake and utilisation of cholesterol, and possibly other lipids, may play an important role in the survival of MM cells.

### Lanosterol

The bone marrow microenvironment (BMME) is an interactive dynamic system that regulates myeloma cell behaviour through different mechanisms. Bone marrow stromal cells (BMSCs) are key components of the BMME, and produce factors, including interleukin-6, B-cell activating factor (BAFF) and a proliferation-inducing ligand (APRIL), that can promote the survival, proliferation and migration of MM cells, thereby contributing to drug resistance [83]. The BMME, especially BMSCs, has been shown to drive ferroptosis resistance through GPX4 deSUMOylation, protecting MM cells from labile iron triggered ferroptosis [84]. A study published in 2024 suggested that BMSCs may also play an important role in the sensitivity of MM plasma cells to ferroptosis through regulation of lanosterol biosynthesis [85]. Lanosterol is a precursor for sterols (including cholesterol) and is readily oxidised, forming ROS in MM cells (Fig. 2) [85, 86]. Co-culture of MM cells with BMSCs sensitised the tumour cells to RSL3 through upregulation of transferrin and iron levels, resulting in iron overload, and lanosterol and cholesterol accumulation [85]. The study also showed that addition of exogenous lanosterol, but not cholesterol, sensitised MM cells to RSL3, while cholesterol alone had minimal effect on RSL3 IC_50_ in their MM cell lines [85]. These results were obtained using MM cells co-cultured with BMSCs, which may explain the difference in cholesterol’s effects observed in this study compared to others that describe cholesterol as an inhibitor of ferroptosis (Table 1).

Importantly, the effects of BMSCs on MM cells were shown to be dependent on cell-to-cell contact and to be mediated by CD40 and its ligand (CD154), as sensitivity to RSL3 was blocked by anti-CD40 antibodies, in both the in vitro and in vivo models studied [85]. This study highlights the important role that sterols may play in the sensitivity of cancer cells to ferroptosis and may represent a potential means of sensitising MM cells to ferroptosis induced by GPX4 inhibition. Interestingly, the study by Jian et al. found that the ferroptosis inhibiting function of BMSCs was also CD40 dependant, suggesting that direct contact between the cells is required to influence ferroptosis [84]. The multifaceted role of the BMME in ferroptosis is complex, and BMSCs may both drive and inhibit ferroptosis in MM.

### Leukocyte immunoglobulin-like receptor B1 (LILBR1)

A recent study identified the leukocyte immunoglobulin-like receptor B1 (LILRB1) as a marker of poor prognosis and a potential target for therapy of MM [47]. Increased expression of LILRB1 is known to play a role in immune suppression [87], but its role in tumour biology has yet to be fully defined. A study by Xian et al.., found that LILRB1-knockdown (KD) in MM cells was associated with an upregulation of oxidative phosphorylation and downregulation of the sirtuin and semaphoring neuronal signalling and antioxidant pathways, which protect cells from oxidative stress [47]. LILRB1 KD also resulted in increased expression of genes involved in fatty acid metabolism and induction of ferroptosis, and decreased expression of genes associated with ferroptosis inhibition [47]. Consistent with these changes, LILRB1 KD significantly increased the sensitivity of MM cells to RSL3, erastin and the ferroptosis-inducer, Fin56, with significantly higher levels of lipid peroxidation observed following treatment [47]. In contrast, MM cells overexpressing LILRB1 were less sensitive to the three compounds, with concomitantly lower levels of lipid peroxidation.

LILRB1 has also been shown to play a significant role in ferroptosis and disease progression in a MM mouse model [47]. LILRB1 KD suppressed disease progression, which was reversed by treatment with the synthetic antioxidant, liproxstatin-1, and RSL3 was effective at reducing tumour burden in a LILRB1 KD, but not control, MM mouse models. Higher levels of lipid ROS were observed both at baseline and following treatment with the GPX4 inhibitor in the LILRB1 KD cells [47]. Consistent with the role of LILRB1 in cholesterol uptake [47], subsequent analysis of LILRB1 KD MM cells from the mice confirmed they contained lower levels of intracellular LDL-cholesterol than MM cells from the control mice [47]. Decreased cholesterol levels in the MM cells from LILRB1 KD mice were consistent with decreased tumour burden, while overexpression resulted in significantly greater tumour burden [47].

### 7-dehydrocholesterol

The precursor, 7-dehydrocholesterol (7-DHC) is converted into cholesterol by 7-dehydrocholesterol reductase (Fig. 2). While there are no studies that investigate 7-DHC in MM, a recent study found that the cholesterol biosynthesis pathway and 7-DHC play important roles in another aggressive B cell malignancy, Burkitt’s lymphoma, as well as neuroblastoma [72]. Contrary to previous research suggesting that 7-DHC is toxic due to its potential to undergo autoxidation, this study found that 7-DHC has pro-survival functions in cancer cells by ‘shielding’ lipids from oxidation, thus preventing ferroptotic cell death [72]. 7-DHC accumulation in Burkitt’s lymphoma xenografts resulted in a more aggressive, ferroptosis-resistant cancer. Addition of free cholesterol to this disease model reduced the protective effects of 7-DHC, suggesting that the balance between cholesterol and its precursors may determine their impact on the sensitivity of cells to ferroptosis. To study the effects of 7-DHC on PL autoxidation in more detail, unilamellar (single lipid bilayer) liposomes loaded with 7-DHC were developed [72]. Using the fluorescence-enabled inhibited autoxidation (FENIX) assay, the authors found that liposomes containing 7-DHC, but not cholesterol, suppressed lipid peroxidation in a dose dependent manner [72, 88]. Furthermore, PL species generated as products of lipid peroxidation were found to be key mediators of ferroptosis [72], which at high levels are also known to activate the intrinsic apoptosis cascade [89]. A separate study of hypoxia-ischemia in the neonatal brain found that elevating 7-DHC levels can suppress ferroptosis and protect against tissue injury caused by an accumulation of ROS [71].

In summary, it is apparent that the cholesterol biosynthesis pathway and levels of the cholesterol pre-cursor, 7-DHC, can modulate the sensitivity of cells to ferroptosis. In addition to 7-DHC, the C27 cholesterol intermediate, desmosterol, has been identified as having anti-ferroptosis properties in human fibrosarcoma and renal adenocarcinoma cell lines [46]. Both cholesterol and desmosterol were found to elevate levels of CoQ10 and squalene, which in turn inhibit lipid peroxidation and protect against ferroptosis (Fig. 2). These effects were also demonstrated in response to treatment with doxorubicin and ischemia-reperfusion induced liver injury in mice [46]. While these studies do not specifically investigate 7-DHC in MM, they do highlight how 7-DHC levels and 7-dehydrocholesterol reductase activity may be associated with prognosis and could be targeted for the treatment of cancers.

### 27-hydroxycholesterol

Perturbations in cholesterol homeostasis have been associated with an increased risk of several cancers, including MM [77, 90]. The cholesterol metabolite, 27-hydroxycholesterol (27HC), has been shown to support tumour cell growth in models of estrogen receptor positive luminal breast cancer [91] and increase metastatic activity in mouse models of breast cancer [92]. Interestingly, tumour burden and the high metabolic activity of the tumour cells in the latter study were shown to be dependent upon inhibition of ferroptosis through sustained expression of GPX4 [92]. This study also concluded that increased survival rates among post-menopausal breast cancer patients receiving statins were also attributable to decreased levels of 27HC [92].

### Sterol regulatory element binding proteins (SREBPs)

Sterol regulatory element binding proteins (SREBPs) are membrane bound transcription factors involved in cholesterol and lipid biosynthesis. SREB2 has been shown to inhibit ferroptosis in melanoma by reducing the labile iron pool and reducing lipid peroxidation by inducing transferrin transcription [93]. A study published investigating artesunate, an anti-malaria drug, has demonstrated promising efficacy in inducing ferroptosis in MM cell lines. Artesunate was able to prevent the nuclear localisation of SREBP2, while GPX4 and IPP were downregulated following treatment, inducing ferroptosis in MM cell lines [94]. ACSL4 levels increased following treatment, which would suggest an increase in PL-PUFA, key cellular substrates of ferroptosis [94]. These results were recapitulated through silencing of SREBP2 using siRNA, which resulted in a decrease in cell viability, an increase in lipid ROS and lipid peroxidation in the MM cell lines [94]. A study by Cai et al.., found that treatment of glioblastoma cells with the SREBP inhibitor, fatostatin, resulted in ferroptosis-mediated cell death via accumulation of lipid ROS and decreased levels of glutathione and GPX4 [95]. Similar results were also observed in a mouse model of glioblastoma using nanoparticles containing fatostatin [95].

B7H3 (also known as CD276) regulates ferroptosis sensitivity in colorectal cancer (CRC) cells by downregulating SREBP2 expression [73]. The knockdown of B7H3 decreased intracellular cholesterol levels, thereby significantly increasing the sensitivity of CRC cells to RSL3 [73]. In contrast, overexpression of B7H3 protects CRC cells against RSL3-induced ferroptosis [73]. The effects of B7H3 knockdown on the sensitivity of the CRC cells to RSL3 were inhibited by addition of exogenous cholesterol. Similar effects were observed in mouse models of CRC [73]. These studies further illustrate the importance of cholesterol and its substrates in determining the sensitivity of cancer cells to ferroptosis.

### Harnessing lipids as a novel therapeutic approach for multiple myeloma

Although ferroptosis was first characterised in 2012 [11], studies that predate this discuss findings that we now know may be attributable to this form of cell death. One such study found that serum levels of the LPLs, lysophosphatidic acid (LPA) and lysophosphatidylcholine (LPC), are significantly elevated in MM patients compared to healthy controls, while levels of lipids containing AA and linoleic acid were found to be lower [96]. AA has been shown to directly induce ferroptosis in MM cells [60], while linoleic acid has been shown to sensitise cancer cells to ferroptosis (studies summarised in Table 1).

Lipid supplementation, particularly with PUFAs, has been investigated in the context of ferroptosis in multiple disease models, including MM and other haematological malignancies (Table 1) [29]. Research has demonstrated that MM cells from patients with specific molecular subsets such as t(4;14) may be susceptible to ferroptosis induction via direct GPX4 inhibition [30]. This study also found that ACLS4 knockdown led to a reduction in PUFA levels and decreased the sensitivity of MM cells to ferroptosis, but that supplementation with even low doses of PUFA (AA or adrenic acid) significantly increased their sensitivity [30]. This study suggests that some high-risk chromosomal abnormalities in MM may respond favourably to ferroptosis induction, warranting further research into other high-risk subtypes such as 1q gain or amplification, and TP53 gene mutation or deletion.

Another study found that supplementing MM cells with either eicosapentaenoic or docosahexaenoic acid, increased cell death via ferroptosis and necroptosis [61]. Interestingly, this study also found that pre-treatment of MM cells with docosahexaenoic acid increased the cytotoxic effects of the proteasome inhibitor, bortezomib, by decreasing cellular glutathione levels [61] These studies suggest that therapeutic approaches targeting multiple cell death mechanisms may be effective in MM.

The cholesterol biosynthesis pathway, involving the substrates lanosterol, 7-DHC, cholesterol, and 27-hydroxycholesterol, are critical determinants of ferroptosis sensitivity in cancer cells (Fig. 2; Table 1). While lanosterol has emerged as a driver of ferroptosis in MM, levels of 7-DHC, cholesterol and 27-hydroxycholesterol have been shown to protect a range of other cancer cells from ferroptosis-mediated cell death (Table 1). As discussed earlier in this review, recent studies have shown that LILRB1, a key regulator of cholesterol metabolism, protects MM cells from ferroptosis by maintaining cholesterol homeostasis [47], while expression of SREBPs are associated with decreased ferroptosis sensitivity in other malignancies [47, 73, 95]. Although the exact roles of SREBPs, which function as transcription factors, and LILRB1, an immune inhibitory receptor, in MM remain unclear, expression of both LILRB1 and SREBPs may decrease the sensitivity of MM cells to ferroptosis by increasing their ability to take up cholesterol [47, 73, 95]. The sensitivity of MM cells to RSL3 following knockdown of these proteins highlights the potential of LILRB1 and SREBPs and the cholesterol biosynthesis pathway as therapeutic targets for MM [47, 73, 95]. However, the published literature concerning the factors that dictate sensitivity of MM cells to ferroptosis, including the role of the BMME, is currently limited. Recent studies suggest that the interaction of MM cells with bone marrow stromal cells [84, 85] and changes in the cellular composition of lipids, including cholesterol [85], that are facilitated by the BMME, play important roles in determining the sensitivity of MM cells to ferroptosis.

Dysregulation of cholesterol metabolism and/or increased uptake into MM cells have been proposed as underlying reasons for the hypocholesterolemia and low serum LPL levels [96], observed in MM patients [75, 80]. This likely plays a significant role in the pathobiology of the disease as low serum cholesterol levels [80] have been associated with an increased risk of MM development [76, 79, 82]. These findings highlight the need for a better understanding of how cholesterol levels are regulated in MM and how this may impact the efficacy of novel, ferroptosis-dependent therapeutic approaches.

In summary, there is growing evidence that ferroptosis has potential as a novel approach for the treatment of MM and a range of other cancers, particularly for patients who develop resistance to apoptosis-dependent regimens. However, there will be significant challenges, including the development of targeted drug delivery mechanisms, before the research findings discussed can be translated into the clinic. Recent advances in nanotechnology-based delivery mechanisms (reviewed in [29]), capable of significantly improving the pharmacokinetics and bioavailability of drugs, have shown promising results. Identifying factors associated with ferroptosis resistance, such as increased levels of 7-DHC or cholesterol, may help to increase the specificity of novel treatment approaches and tailor therapies to individual patients.

## Conclusions

Multiple myeloma is the second most common haematological malignancy in Australia and worldwide. The management of MM patients is challenging due to clinical and biological heterogeneity between patients and, despite advances in therapy over the past few decades, it is still considered incurable. A significant proportion of patients will relapse with drug-refractory disease, necessitating the development of novel therapeutic approaches. Ferroptosis, a relatively recently defined mechanism of programmed cell death, represents a promising approach for the treatment of MM and other cancers, that are refractory to apoptosis-mediated regimens.

However, a better understanding of the relationship between cellular lipid composition and ferroptosis sensitivity is key to harnessing this form of programmed cell death as a therapeutic approach. Studies to date indicate that cholesterol levels impact not only the onset and progression of haematological malignancies but also the sensitivity of a variety of different cancers to ferroptosis. Therefore, targeting the metabolism and uptake of lipids, including glycerophospholipids and cholesterol may be an effective means of sensitising tumour cells to ferroptosis-inducing drugs and could offer new strategies to treat patients who are resistant to more conventional, apoptosis-dependent treatments. Findings from the limited number of studies concerning ferroptosis in MM and compelling evidence from other malignancies, provide a strong rationale for further investigation of ferroptosis as a novel therapeutic approach for MM.

## Data Availability

No datasets were generated or analysed during the current study.

## Abbreviations

27HC: 27-hydroxycholesterol
7-DHC: 7-Dehydrocholesterol
AA: Arachidonic acid
Acetyl-CoA: Acetyl coenzyme A
ACSL4: acyl-CoA synthetase long-chain family member 4
BMME: Bone marrow microenvironment
BMSC: Bone marrow stromal cell
CoQ10: Ubiquinone
CoQ10-H2: Ubiquinol
FA: Fatty acid
FPP: Farnesyl phosphate
FSP1: Ferroptosis suppressor protein 1
GGPP: Geranylgeranyl pyrophosphate
GPX4: Glutathione peroxidase 4
H_2_O_2_: Hydrogen peroxide
HDL: High-density lipoprotein
IPP: Isopentenyl phosphate
HMG-CoA: 3-hydroxy-3-methylglutaryl-coenzyme A
LDL: Low-density lipoprotein
LILRB1: Leukocyte immunoglobulin-like receptor B1
LOX: Lipoxygenases
LPC: Lysophosphatidylcholine
LPCAT3: Lysophosphatidylcholine acyltransferase 3
LPE: Lysophosphatidylethanolamine
LPL: Lysophospholipids
MM: Multiple myeloma
MMSET: MM SET domain-containing protein
MUFA: Monounsaturated fatty acid
OH^−^: Hydroxide ion
PC: Phosphatidylcholine
PCD: Programmed cell death
PE: Phosphatidylethanolamine
PL: Phospholipid
PL-PUFA-OH: Phospholipid alcohol
PL-PUFA-OOH: Phospholipid hydroperoxide
PS: Phosphatidylserine
PUFA: Polyunsaturated fatty acid
ROS: Reactive oxygen species
RSL3: (1S,3R)-RSL3
SFA: Saturated fatty acid
SREBP: Sterol regulatory element binding protein

## Declarations

### Availability of data and materials

There is nothing to declare.
